# Perspectives from Young Australian Women with Lived Experience on Why Rates of Self-Harm Are Increasing: A Qualitative Study

**DOI:** 10.3390/ijerph22121871

**Published:** 2025-12-16

**Authors:** Lorna Hankin, Anastasia Hronis, Alexis Whitton, Samantha Tang, Aimy Slade, Helen Christensen, Alison L. Calear, Katherine Boydell, Demee Rheinberger

**Affiliations:** 1 Discipline of Clinical Psychology, Graduate School of Health, University of Technology Sydney, Ultimo, NSW 2007, Australia; 2 Black Dog Institute, UNSW Sydney, Randwick, NSW 2031, Australia; 3 Faculty of Medicine and Health, UNSW Sydney, Kensington, NSW 2052, Australia; 4 Centre for Mental Health Research, The Australian National University, Canberra, ACT 2601, Australia

**Keywords:** self-harm, non-suicidal self-injury, young women, social media, qualitative research

## Abstract

Rates of self-harm in Australian young people have increased significantly in recent years, especially in young women. Self-harm has been associated with several risk factors, including a history of abuse, bullying, mood and personality disorders, social isolation and suicidal ideation. However, little is known about why rates have increased in the past decade, and the voices of young Australian women have been conspicuously absent from the research. This study explored perceived subjective reasons for the increase in self-harm rates by interviewing 24 young Australian women with lived experience of self-harming behaviours. A reflexive thematic analysis identified three interwoven themes: ‘The world is hard, and it’s getting harder’, ‘New media exacerbates old challenges’, and ‘The online world brings unique challenges’. Participants also highlighted the complexity of social media as both a negative influence and a supportive factor. These themes extend previous research by highlighting the nuanced and multi-faceted psychosocial factors that influence self-harming behaviours and may help inform effective, evidence-based strategies that help minimise harm.

## 1. Introduction

Self-harm, intentional injury or damage to oneself, with or without intent to die, continues to be of considerable concern, particularly among young women [1]. Research consistently indicates that rates of self-harm are higher in young women than in young men [2,3,4,5]. In Australia, self-harm-related hospitalisations for 15–19-year-old women rose by almost 87% in 2021–2022, [6]. However, rates of intentional self-harm hospitalisations in females aged 14 years and younger have increased more than three-fold between 2008–09 and 2022–23 [6].

A significant body of research suggests self-harm serves both intrapersonal (emotion regulation or management) and interpersonal (social) purposes and can lead to positive emotions and validation of identity [1,7,8,9]. Studies have also explored the risk factors for self-harm, which include a history of abuse, bullying, mood and personality disorders, social isolation and suicidal ideation [10,11,12,13]. However, there is limited research exploring possible reasons for recent increases in self-harm prevalence, particularly in young women [5].

It has been posited that rapidly changing social and economic conditions may have increased stress and pressure on young people leading to an increased risk of self-harm [1,14,15]. In the past decade, young people have been exposed to a global pandemic, multiple international conflicts, growing rates of social economic disadvantage and intergenerational inequalities, and concerns about the rapidly increasing effects of climate change, all of which may have contributed to overall increasing rates of mental ill health in young people [16,17]. A further body of literature focuses on possible correlations between digital media, including the internet, smartphones and social media platforms, and increases in rates of self-harm and other mental health conditions in young people [18,19,20,21,22]. Specifically, some researchers have highlighted apparent correlations between identified increases in self-harm rates and digital events such as the introduction of the iPhone and Instagram, noting that these events may have led to reduced face-to-face interaction and sleep quality, and increased exposure to cyberbullying, ‘toxic’ environments, and information about self-harming behaviours [21,23]. Correlations have also been identified between increases in self-harm rates and Netflix shows such as *13 Reasons Why*, which researchers have suggested might be due to the effect of social contagion or due to explicit content increasing distress in viewers [24,25]. It has also been suggested that social media specifically may have a greater negative effect on young women than on young men, which some researchers posit may be due to higher social media usage and more frequent exposure to negative social comparisons, particularly regarding body image [21,22,23].

Although this research has garnered significant media attention and had an influential role in the Australian government’s recent decision to ban social media for young people under 16 years old, an opposing body of research has critiqued these findings [26]. Researchers suggest that studies drawing associations between technology use and mental ill health, including self-harm, lack consensus and transparency [26,27,28,29,30]. Specifically, associations reported in longitudinal studies and meta-analyses are often too small to be clinically significant [26]. Moreover, many correlational studies are cross-sectional and include secondary analyses of data that were not collected for the purpose of studying social media, thereby significantly increasing the risk of publication bias [31,32]. Importantly, the voices of young people regarding impacts of social media on mental health have also been conspicuously absent in the research [33].

Similarly, there is a paucity of literature examining the wider views of young women regarding what they think is driving the prevalence of self-harm [1,5], and most qualitative research has been conducted outside of Australia [11,34,35,36,37]. To support young people with effective, evidence-based strategies that help minimise harm, it is essential to understand all the factors that may be driving the increase in self-harming behaviours [5]. Moreover, to understand the phenomenology of self-harm, the perspective of young people with lived experience is critical [1,38]. To date, no studies have examined perceived factors driving the increase in self-harm in young Australian women, nor have they directly asked young women who have engaged in self-harming behaviour for their views on why self-harm may be becoming more common. Therefore, this study aimed to examine the perceived reasons for the increase in self-harm prevalence rates from the perspective of young Australian women with lived or living experience of self-harm.

## 2. Methods and Materials

This qualitative investigation is part of a wider qualitative study which explored the experience of self-harm among young women, the findings from which will be reported across multiple papers (e.g., see [39]). This research was approved by the UNSW Human Research Ethics Committee (HC230437). Informed consent was obtained from all study participants.

The research was guided by a relativist ontological stance, which acknowledged that the realities of the young women are diverse and informed by their own contexts. In alignment with constructionist epistemology, which recognises that meaning is created through contextual understanding and interpretations, a reflexive thematic analysis approach [40] was utilised.

### 2.1. Recruitment

Participant recruitment was conducted between October and November 2023. Purposive sampling was utilised via study advertisements on Black Dog Institute’s (BDI) social media platforms, including Facebook, Instagram, X (formerly Twitter) and LinkedIn. After engaging with the study advertisements, potential participants were directed to a study website with information about the study and a link to the Participant Information Statement and Consent Form. After providing informed consent, individuals were directed to a short screening questionnaire to determine eligibility to participate in the interviews. Individuals were eligible if they identified as female, were 16–24 years old, lived in Australia, could speak and understand English clearly and endorsed previous or current deliberate self-harm, defined as “intentionally causing pain or damage to their own body (either with or without suicidal intent)”. Young women who indicated that they were aged 16 or 17 years were also required to complete and pass a Gillick Competency Task [41], which ensured that they had sufficient maturity to understand study requirements. Eligible participants were asked to provide their name; demographic information, including age, employment status, mental health history and contact details; and available times and dates for interview. Ineligible participants were directed to a page containing support helpline contact details. Sample size was predetermined based on Malterud’s concept of information power [42] which recommends a moderate–large sample size to ensure an in-depth investigation of individual experiences of the broader topic of self-harm, as such the study aimed to recruit between 25 and 30 participants.

### 2.2. Procedure

Eligible participants attended a one-on-one online interview conducted via secure video conferencing software (Zoom) (version 5.15.5). Interviews were conducted between November and December 2023 and ran for approximately 60–90 min (average: 68 min). Interviews were conducted by one of five Clinical Psychology Masters students who were also provisionally registered psychologists (including LH). The semi-structured interview guide was collaboratively developed by members of the research team and two young women with lived experience of self-harm during a workshop in which topics for examining why young women are engaging in self-harm were brainstormed and prioritised. The topics were then examined by the research team to develop a set of interview questions, with priority given to those deemed the most important during the workshop. These interview questions were circulated to all workshop participants for feedback and resulted in the final interview guide (see Supplementary File S1).

Interviewers followed a safety protocol to respond in case of a participant’s disclosure of imminent risk of suicide. If a participant indicated they engaged in self-harm with suicidal intentions, interviewers were to ask follow-up questions to ascertain the presence of immediate risk of suicide. If there were concerns for the participants safety interviewers were to facilitate immediate intervention by a supervising clinical psychologist to ensure participant safety. Since all interviewers were registered psychologists, they were also encouraged to utilise their clinical judgement and pause or cease the interview should a participant show considerable distress during the interview process. Such participants would also be connected with appropriate clinical support if necessary. No participants indicated imminent suicide risk or became so distressed that interviews were terminated early.

At the end of the interview, participants were provided with information about support helplines, offered the opportunity to be contacted by a registered Clinical Psychologist, and were provided an e-gift card for their participation which was offered as an acknowledgement of participants’ time and effort required to prepare for, attend and complete the interview, as well as a deeper acknowledgement of the value their story is bringing to the research. Audio recordings and transcripts were de-identified and sent to a third party for transcription. Participants were not provided with a copy of the transcript for review.

### 2.3. Analysis

Twenty-seven interview transcripts were imported into NVivo software (version 12) for analysis. Participant responses to a single interview question: “Research has shown that more young women are self-harming now than 10 years ago. Do you have any insights into why that would be?” form the basis of the current analysis. Twenty-four participants (89%) provided a response to this question.

Participant response data was analysed using reflexive thematic analysis (RTA) [40] to uncover and consider common themes and patterns of meaning embedded in participant narratives. Two researchers (LH and DR) applied the six RTA steps using established guidelines [40]: 1. Data familiarisation (researchers independently immersed themselves in the dataset, re-reading the transcripts and noting first impressions and initial ideas); 2. Generating initial codes (researchers independently and systematically worked through the data to identify and interpret information relevant to the research question, meeting at regular intervals to discuss and further develop codes); 3. Generating themes among codes (researchers identified broader topics and patterns of meaning within the codes, to capture the essence of the data); 4. Reviewing potential themes (researchers revisited and reassessed themes, collapsing or dividing some themes where necessary, and continuing to meet to discuss their reflections on the data and to discuss repeated patterns of meaning); 5. Defining and naming themes (researchers developed clear and concise names that captured meaningful and distinct aspects of the data); and 6. Write-up (both researchers took active roles in the preparation of the manuscript).

Code and theme generation was informed by the researchers’ understanding of the literature relating to motivations and precipitating factors which have long been associated with self-harm behaviour. Researchers built on this understanding during the theme generation which facilitated the grouping of codes into themes which demonstrate both previously examined self-harm correlations and the impact of the modern world.

The process of developing codes and themes was flexible and evolved throughout the analytical process, with further data familiarisation leading to new interpretations and patterns of meaning [40,43]. Researchers (both female) comprised LH, who is a Clinical Psychology Masters student with experience engaging with clients who have engaged in self-harm and DR, a researcher who has extensive experience conducting qualitative suicide and self-harm research. Both researchers viewed self-harm as a symptom of emotional distress which is exacerbated by challenging environments and social factors, and which can be addressed through psychological, situational, and social interventions. LH and DR both believe the reasons why a young woman chooses to engage in self-harm is unique to the individual but recognise the well-established evidence of motivations and precipitants which typically inform self-harm behaviours. Reflexive memos were kept to inform the analysis process and to allow the researchers to understand how their own world views may have informed conclusions drawn from the data. Final themes were arrived at through in-depth discussions between the two coders and a third researcher (AH, a clinical psychologist) who was independent from the coding process. Research rigour was maintained throughout the analysis process through a team approach to the analysis, maintenance of a detailed audit trail and reflexive notes, and through prolonged engagement with the topic under investigation. This manuscript has been reported in accordance with the COREQ Checklist (see, Supplementary File S2).

## 3. Results

### 3.1. Participant Demographics

Twenty-four participants provided a response to the interview question under investigation. Participants mean age was 20.92 years (*SD* = 2.1; range = 18–24 years). All participants had engaged in self-harm behaviours on more than one occasion, with 58% describing their self-harming behaviours as ‘current’ (*N* = 14; missing = 3). All participants had received mental health diagnoses. No participants identified as Aboriginal or Torres Strait Islander. Detailed participant demographic information can be found in Table 1.

### 3.2. Thematic Analysis

**Figure 1:**
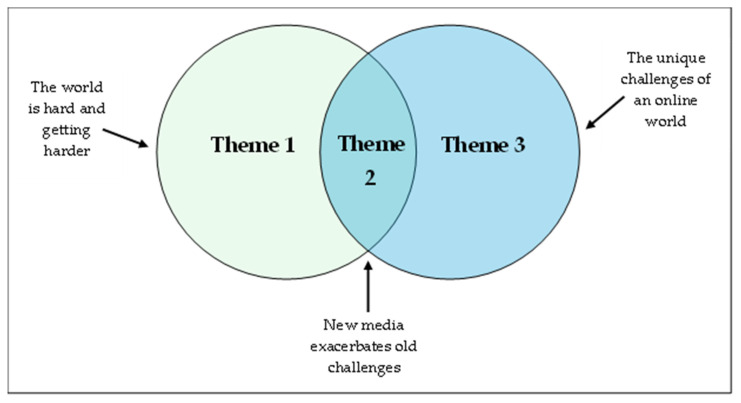
Interwoven themes.

Thematic analysis resulted in three interwoven themes (Figure 1), which highlighted that the factors that participants perceived may be driving self-harm rates are both inherent within our society (present over numerous decades) and also being rapidly influenced by an evolving online world. This is explored within the three themes.

First, participants described influencing factors that have always existed and may be becoming more challenging (The world is hard, and it’s getting harder). Second, participants described influencing factors that have always existed but that are now being exacerbated by the online, digital world (New media exacerbates old challenges). Third, participants described influencing factors that are unique to the online world (The online world brings unique challenges). A summary of the themes and related codes can be found in Table 2.

#### 3.2.1. Theme 1: The World Is Hard, and It’s Getting Harder

In response to the question of why they believe rates of self-harm are increasing, many participants began by describing “old world” challenges. That is, factors that research has identified as increasing the risk of self-harm over previous decades. This includes complex friendship dynamics, a sense of hopelessness or helplessness driven by world events, feeling unsupported or misunderstood by caregivers and medical professionals, and feeling pressured to look and act in a certain way.

Some participants spoke of the complexity of dynamics within friendship groups, which could lead to intense connections that have powerful influences on members’ behaviours. For example, while some participants acknowledged that such close friendship groups afforded them a sense of connection, they also sometimes introduced unhelpful coping mechanisms.

*“…it just kind of spreads to other people within close female friendships…you think of their coping mechanism like it’s yours or something. There’s this feeling of an understanding or you want to be close to your friends.” *

[Participant 10]

*“They can be really supportive because you feel understood, but I think ultimately people can end up dragging each other down…where every person is struggling with mental illness it can actually be detrimental.” *

[Participant 1]

Participants also commented on the ongoing pressure young women face to look, behave and perform in a certain way—physically, socially, and academically—which is embedded in our cultures.

*“A majority of girls get some serious mental health struggles from the way that their bodies are valued or devalued depending on their environment. Because it’s 2023 and a woman is still valued for her body and how well she can make babies.” *

[Participant 6]

Moreover, in many instances, participants posited that rates of self-harm were increasing in response to “old world” factors becoming more prevalent and expectations more intense.

*“The HSC *[high school certificate] *is a hugely stressful thing to go through, and I think the pressures are increasing. I’ve looked at the syllabus, there’s always stuff being added. The stuff I was doing in physics for year 12 was stuff that five to 10 years ago would’ve been in third, fourth year university.” *

[Participant 18]

For some participants, external societal pressure was internalised, leading to self-harm acting as both a pressure outlet and a punishment if expectations were not met.

*“I think the landscape, especially for education, has become so competitive and increasingly so over the past decade, and so when there’s just no room for error, self-harm can be…an expression of perfectionism and excessive self-control and intolerance of mistakes.” *

[Participant 15]

Notably, many of the participants identified the impact of a world facing an increasing number of crises, commenting *“the world’s shit, the world’s terrible” [Participant 11]* and *“the world kind of sucks right now.” [Participant 8]*

Similarly, participants felt a general sense of hopelessness or helplessness in the face of international events. “For people of my age…we’ve been born into global discussions: It’s COVID, or it’s global warming, or it’s this f*cking war that’s going on in Palestine, it’s just shit after shit. And I think that that can really take a toll on you.” [Participant 11]

Participants also consistently commented on crises that were closer to home as possible driving factors of increased rates of self-harm.

*“Obviously cost of living, all that stuff that has a big impact on people. Housing and all of those things.” *

[Participant 24]

Several participants directly highlighted the impact of COVID-19 and a sense of social isolation. *“We all felt so alone for so long. It’s a tricky thing to navigate…how are we meant to learn this formative stuff on our own with no social connection.” [Participant 23]*

Worryingly, despite the number of challenges they faced, participants also identified that it is very difficult to get affordable, timely, and appropriate mental health support. This left some feeling misunderstood by caregivers and medical professionals and needing to rely on their peers.

*“You can’t get into GPs for weeks and you can’t get into psychologists for a month and then you’ve only got 10 sessions on Medicare…they’re not even fully covered for an entire year.” *

[Participant 8]

*“…they would always say, reach out to your trusted adult. And sometimes that’s just not there…so I think that children are relying on each other, and it can be quite dangerous.” *

[Participant 17]

This theme suggests young women with a lived experience of self-harm believe that some of the factors which lead to self-harm behaviours in young women may be similar to those reported in previous decades. This includes friendship difficulties, considerable pressures to perform well academically, and social expectations for how women should look and behave. Young women indicated that it is still difficult to access support from trusted adults and healthcare professionals. Young women also discussed the impact of global crises and their increased sense of worry and despair as a result and posited these global crises may increase self-harm behaviours. Finally, young women proposed that the lack of support provided by caregivers and healthcare professionals could exacerbate negative feelings and increase self-harm rates.

#### 3.2.2. Theme 2: New Media Exacerbates Old Challenges

There was a strong sense from participants that rates of self-harm may be increasing because not only were “old world” factors still prevalent and potentially getting worse, but also that the online world has greatly amplified these concerns.

First, participants described the increase in general awareness of self-harm that the online world provided, and the impact that could have on already vulnerable young women.

*“I think the rates at which people are online and engaging with content that isn’t appropriate for them is probably going up. Access has really increased for young people…And I think if young people see that concept and it’s what they’re looking for at the time…young people [who] are mentally ill and distressed and they hate their bodies and have poor self-image and they’re looking for that, then they’ll engage in it.” *

[Participant 20]

Others noted that this increased awareness acted as a double-edged sword, both helping to reduce stigma, while exposing young women to confronting content without accompanying education or support.

*“I think when you’re seeing it [self-harm] a lot more and when something is a lot more normalised, it’s the first stage towards discussing something and making it not quite as taboo. It’s necessary…but I think at the same time, when you expose a great deal of people to a topic without much education around it externally, I think that’s where you have issues.” *

[Participant 22]

Participants also noted that online communities made it easier for young women to become immersed in friendship groups that engaged in self-harm and may influence similar behaviours.

*“I think there’s also more ways for young people to engage in communities that revolve around those maladaptive behaviours. It’s a lot easier now than it would’ve been [a decade ago] to go online, find a group or a forum where people are actively encouraging each other, giving each other little tips and tricks…” *

[Participant 20]

There was also a sense that the online realm increased awareness of the world in crisis and exposure to traumatic world events.

*“I definitely think the internet, I definitely think the state of the world is quite anxiety inducing. Everything is becoming more stressful, and the internet is pushing that stress onto everyone.” *

[Participant 10]

This theme explored the perception that the online world exacerbates the negative impacts of preexisting factors which influence young women’s mental distress or decisions to engage in self-harm. This included increased exposure to greater volumes of information about international crises and world events, and the stigmatising opinions of others. Furthermore, access to more information about self-harm and to online communities which could be seen to encourage self-harm behaviours may further exacerbate such behaviours.

#### 3.2.3. Theme 3: The Online World Brings Unique Challenges

Participants proposed that the increase in self-harm among young women may be result of a set of challenges that are unique to the online world, including unlimited access to social media platforms, streaming services and algorithm-driven content. These are new challenges which are experienced as a result of the online world, rather than pre-existing challenges heightened by digital media (which were discussed in theme 2).

The most common response to the interview question was the rise of social media, particularly visual platforms such as Tumblr, Instagram and TikTok: *“I think social media is a big influence [on self-harm rates], especially TikTok. It definitely was for me.” [Participant 19].* Some participants commented specifically on how accessible and unfiltered self-harm-related content was on social media: *“A lot of information is very readily available, and not all of it is positive…all unfiltered social media is going to be an issue when it comes to that.” [Participant 22]*

Others felt that not only did social media increase awareness of self-harm, but it encouraged comparisons and “glorification” in ways that were not previously possible.

*“It’s just so much easier to see a range of different experiences these days than it was 10 years ago. You can find self-harm material online, you can find self-harm communities where people compare themselves to each other…10 years ago, if someone was in your class who was self-harming, that was probably how you heard about it…[but] I grew up in the Tumblr era, and there was a lot of self-harm glorification there.” *

[Participant 27]

One participant noted that even when communities appeared to be a place of acceptance, comparison was rife: *“During my rough periods, I saw some of the stuff that’s on Twitter and Reddit, and it’s really horrific. I think even during that time, I was like, this is in no way helpful for anyone…it was very much, almost like a competition between who was suffering the most.” [Participant 22]*

Participants also commented on the role of powerful algorithms across platforms in immersing people in increasingly distressing content: *“Especially things like TikTok where once you see one thing and then the algorithm just hones in on it and starts giving you more and more and more of the same thing.” [Participant 2]*

Social media platforms were not the only factors unique to the online world that played a part; participants also highlighted the role of streaming services (such as Netflix) in potentially leading to an increase in self-harm rates. Streaming services and online video platforms make it increasingly easy to access television shows and movies, such as Netflix’s *13 Reasons Why*, which depict self-harm and suicidal ideation. Participants noted this often led to more people engaging in the depicted self-harm behaviour.

*“I remember seeing a statistic that self-harm research showed that rates of young girls self-harming skyrocketed after *13 Reasons Why *came out and I’m like, this is what I’m talking about.” *

[Participant 6]

More broadly, participants felt that the online world had influenced self-harm rates by impacting mental health. One participant commented: *“I would stay up late on my phone talking [online] to my partner and trying to convince them not to harm themselves and that affected my sleep, which affected my mental health.” [Participant 1]*

Others highlighted the deceptive nature of social media, which could fuel unhelpful social comparison and a *“feeling of loneliness and sort of inadequacy.” [Participant 4]*

*“With filters, everything’s so deceiving. It makes you think, why am I not looking like that? Whereas in reality, no one looks like that.” *

[Participant 12]

*“It’s like, why are these people out partying every weekend? Does this mean no one likes me? But it doesn’t, they’re not actually out partying every weekend. They’re just making it look like that.” *

[Participant 23]

This was compounded by some participants’ views that the online world reduces their ability to regulate emotions and increases a sense of pressure to be busy and ‘always on’; *“There’re no more micro moments of quietness, micro moments of boredom. We can’t tolerate boredom anymore. We’re just too busy. We’re too distracted…There is that decrease in capacity for a human experience almost, which leads you to taking shortcuts and that short-term relief.” [Participant 13]*

Participants also noted that these factors were becoming a problem at an increasingly young age.

*“Kids nowadays are getting a tablet by before they turned 10, they have access to the internet that whether or not they know will shape their minds…I didn’t have a phone that I had social media to scroll on until I was 13. I didn’t have Instagram until I was 16. Nowadays the access to the internet is so easily accessible, which is not exactly a good thing.” *

[Participant 12]

Importantly, participants also highlighted the role of the online world in lowering rates of self-harm by reducing stigma, encouraging people to seek help and providing a place of belonging.

*“I would say it can impact it negatively or positively depending on which corner of the internet you end up in… Personally, when I was younger and first figuring out I was queer, the internet and queer communities on social media was all I had, so it was very beneficial.” *

[Participant 8]

Others wondered if rates had really gone up, or if the online world meant that people just felt more *“comfortable coming forward with their self-harm issues rather than completely hiding them.” [Participant 18]*

*“I think part of it could be just about how it’s recorded, how they find the data. 10 years ago, I’m pretty sure it would be much more difficult to collect this sort of data. More people would be much more reserved about mental health.” *

[Participant 9]

In summary, the young women reported that the algorithms and increased exposure to self-harm depictions on social media had the potential to influence rates of self-harm. Similarly, online communities fostered through social media sometimes encouraged community members to engage in more risky self-harm behaviours. Furthermore, the young women discussed how the online world can negatively impact mental health by increasing social comparison and disrupting sleep hygiene. Additional young women spoke about the cognitive impacts of constantly being online, and the absence of boredom as a result. However, the young women also theorised that self-harm rates might be increasing due to higher rates of reporting, as a result of reduced stigma and greater mental health education, which is often accessed online.

## 4. Discussion

To support young people with evidence-based strategies that help reduce incidences of self-harm, it is essential to understand factors driving recent increases in self-harming behaviours. This study aimed to build on the emerging body of literature by seeking the perspectives of young Australian women with lived experience of self-harm, who are often left out of the conversation [33]. This is the first study to examine these perspectives and to examine the influence of the online world on how young women experience preexisting and new precipitants to self-harm. Three interwoven themes were identified describing factors that participants considered may be influencing the increase in prevalence: 1. ‘The world is hard, and it’s getting harder’, including the intensity of friendship groups, the pressure on young people, a lack of support and the world in crisis; 2. ‘New media exacerbates old challenges’, including increasing awareness of and exposure to self-harm content and related communities, and the state of the world; and 3. ‘The online world brings unique challenges’, including algorithm-driven social media platforms and streaming services, which can encourage comparison and negatively impact mental health, while also reducing stigma and providing support. These themes align with previous research that reflects the complex and multi-faceted psychosocial factors that influence self-harming behaviours [1,37].

These findings are in alignment with Nock’s Four-Function Model (FFM) of Non-Suicidal Self-Injury, which suggests that the reasons for an individual to engage in self-harm can be mapped to four reinforcement processes (to either increase or decrease emotional or social states) [44]. For instance, participants spoke of access to online communities which further encouraged self-harm behaviours (positive social reinforcement) and of engaging in self-harm in response to distress which is due to ongoing and overwhelming reporting of world crises (negative automatic reinforcement). These findings provide evidence that the FFM applies to online experiences as well as those which have been long standing issues for young people who self-harm.

### 4.1. Theme 1: The World Is Hard, and It’s Getting Harder

The challenges highlighted in this theme build on the extensive literature highlighting how established risk factors remain an influence on self-harming behaviours in young women, including interpersonal connections within friendship groups [45], societal and academic pressure [1,46] and a lack of understanding from caregivers and health professionals [37]. In many instances, these aspects may be worsening; for example, academic pressure has increased [46], and availability of subsidised psychological support has declined [47].

This study adds to a growing body of research that highlights the long-term impact on young women of challenging world events [1,16]. This is perhaps unsurprising given the past 10 years have seen a global pandemic, the persisting climate emergency, the current cost-of-living crisis and increased political unrest [16]. Previous research has suggested that young people with high levels of climate change concerns are more likely to report mental ill health, although the association is likely to be bidirectional [48]. Moreover, participants identified that events such as the COVID-19 pandemic may have impacted self-harm prevalence by increasing a sense of social isolation, which is also a known risk factor for self-harming behaviours [11,12]. The current literature exploring whether incidences of self-harm increased during COVID-19 lockdowns is mixed, although research increasingly highlights the impact of the pandemic on overall mental health [14,15,17]. Importantly, research suggests that global challenges can have a disproportionate impact on young women, due to their increased risk of economic hardship, education and employment instability, and higher rates of gender-based violence during economic and political unrest [26].

### 4.2. Theme 2: New Media Exacerbates Old Challenges

The emphasis on the impact of a world in crisis was also apparent as participants highlighted the power of online media to raise awareness of traumatic world events and subsequently rates of self-harm. Previous research suggests that access to online depictions of traumatic events, such as war, terrorism and COVID-19, is associated with higher psychological distress [49,50], as well as higher post-traumatic symptoms in adolescents than depictions in traditional media [50]. It is possible that young people could inadvertently become engaged in algorithms that excessively expose them to this content, highlighting the need for policy makers and technology companies to ensure that safety measures are in place for young people in the online space. For example, balancing depictions of traumatic events with online content showing related acts of kindness may mitigate distress [49]. Additionally, young people would benefit from education which enables them to critically evaluate their social media use, and algorithmic decision-making powers [51].

Participants also suggested that increased access to the internet at a younger age, and to online media more broadly, brought a new awareness of, and exposure to, self-harm-related content, as well as increasing the opportunity for young women to form connections with others who engaged in self-harm in online communities. This may support the view that increased exposure to depictions of self-harm as an adaptive or culturally endorsed method of managing emotions can increase self-harming behaviours through social learning [1]. Moreover, despite the potential to reduce stigma, participants in this study suggested increased exposure to online content and communities may be particularly problematic as it is not currently accompanied by an increase in education or support, underscoring the need for an increase in preventative resources that directly address the issue and that effectively meet the needs of young people at risk of self-harm [5,52].

### 4.3. Theme 3: The Online World Brings Unique Challenges

Theme three emphasised both the influence that the online world, and social media specifically, may have on self-harm rates and the need for a nuanced response to this possibility. Participants commented that highly visual social media platforms, driven by powerful algorithms, could immerse young women in self-harm-related content that inspired glorification, comparison and competition. This supports a growing body of research suggesting a correlation between social media use and self-harm [22,23], including findings that online visual depictions of self-harm may trigger or escalate self-harm episodes or inspire greater wound severity [19,20]. Participants also felt that the online world had influenced self-harm rates by impacting mental health, including increasing loneliness and social comparison, and reducing people’s ability to regulate their emotions. Indeed, 42% of young Australians cite social media as the main reason for a decline in their mental health [53]. Social media use may also be linked to a reduced ability to tolerate and regulate emotions [54], which in turn may be associated with self-harm [7]. Beyond social media, participants also commented on the impact of streaming shows such as *13 Reasons Why,* aligning with research that showed a significant increase in self-harm-related visits to emergency departments after the show’s release [24,25] and highlighting the need for further research into the impact of cultural messaging on young women’s beliefs about self-harm [1].

However, the direction of the correlations between social media use, mental ill health and self-harm in young women are still far from certain [1]. Longitudinal studies and meta-analyses often report significant methodological weaknesses and associations which are too small to be clinically significant [1,26,31,32]. Moreover, other qualitative research suggests young women who access self-harm related content online are likely to already be self-harming [55]. This highlights the pressing need for additional research investigating causal rather than correlational relationships and further exploring alternative explanations for an increase in self-harming behaviours, specifically in young women [1,26,28,31,32].

Crucially, participants also highlighted the ‘double-edged sword’ of the online world [56], noting its ability to reduce stigma, encourage help-seeking and provide a place of belonging. This aligns with research highlighting the role of online spaces in providing young people with a sense of social connection, safety and support [1,27,28,30]. Moreover, minority groups, including neurodivergent, gender diverse and sexual minority youth, as well as young women, have reported the benefits of accessing mental health and community support online, which is particularly important given the increased risk of mental ill health, self-harm and suicide in these cohorts [1,26,55,57]. Given recent Australian policy which will ban individuals younger than 16 years of age from accessing social media [58], further research is urgently required to understand the risks versus benefits of online support communities, including those that provide support for young women who self-harm, as well as alternative ways of mitigating risks, before social media bans take effect.

### 4.4. Strengths and Limitations

This study has a number of strengths, including a large sample size, consistency in responses, a rigorous analysis process, and the novelty of the research question; this is to the authors’ knowledge the first time young women have been asked for their perception of the increase in rates of self-harm. However, this also meant participants’ responses to this question were based on subjective opinions and their viewpoints may be influenced by consumption of media that says social media is causing rising self-harm rates [59].

The study was also impacted by further methodological limitations. First, the online recruitment strategy may have prevented young women in low social economic areas, or from Aboriginal and/or Torres Strait Islander background, without consistent access to the internet or social media from contributing [60], despite higher rates of self-harm in both these populations [1,4]. This removed the opportunity for exploring cultural factors that influence self-harm and limited generalisability. Second, while there is considerable evidence to suggest that a large proportion of young Australians are online/utilise social media regularly [61], the online recruitment strategy may have resulted in participants who are more likely to be engaged online and more likely to seek online support. This may influence the generalizability to young women who do not use social media. Additionally, the absence of younger participants (aged 16 and 17 years) could have been impacted by the recruitment strategy which did not advertise on social media platforms which are more popular with adolescents, such as TikTok [61]. Third, the absence of 16 and 17 year olds in the study may have limited the presence of developmental factors in self-harm such as identity formation and emotion regulation which have been demonstrated in the wider literature [62,63]. Future research should seek to include these groups. Fourth, since the research question examined in this manuscript was one of several aims of the interviews, some nuance and depth may be missing from participant responses. In spite of this, we did identify text that was illuminating of the reasons why, from the perspective of the interviewee, rates would be increasing. Furthermore, the research question was asked at the end of the interview and because of time restraints, or participant fatigue, some individuals were not provided the question or may have not wanted to give much detail in response. A more in-depth investigation of this topic could provide more insights, including a longitudinal approach to better capture changing risk factors over time. Finally, the results are focused on a particular cohort of young women and future researchers might explore specific subgroups of young women, or young people more broadly.

## 5. Conclusions

In an increasingly divisive research landscape, this study was the first to ask young Australian women with lived experience for their views on why rates of self-harm are increasing. Their responses emphasise the multi-faceted psychosocial factors that influence self-harming behaviours, including the increasing complexities presented by the online world. The study was permeated by the sense that the world is becoming more stressful, and that the internet is increasing that stress. Social media in particular creates an environment which can exacerbate traditionally ‘offline’ challenges such as access to self-harm depictions. Nevertheless, many of the factors that participants highlighted are well-studied precipitants of self-harm, suggesting future research should not lose focus on supporting young women with these ‘offline’ challenges. Future research should explore the importance of better social media governance and education in reducing incidences of ‘toxic’ content and environments, consider how best to retain the sense of connection and support the online world provides for young women who already feel isolated and marginalised, and support them to develop the emotion regulation and critical thinking skills required to navigate its content and communities.

## Figures and Tables

**Table 1:** Participant demographic information.

Demographic		n (%)
Employment status	Employed part-time	18 (75.0%)
	Unemployed	6 (25.0%)
Student status	Student full-time	10 (41.7%)
	Student part-time	7 (29.2%)
	Not studying	7 (29.2%)
State or territory of residence	NSW	9 (37.5%)
	QLD	5 (20.8%)
	VIC	5 (20.8%)
	SA	2 (8.3%)
	TAS	2 (8.3%)
	Other *	1 (4.2%)
Mental health diagnosis **	Depressive disorder	18 (75.0%)
	Anxiety disorder	14 (58.3%)
	Feeding and eating disorder	7 (29.2%)
	Neurodevelopmental disorder	5 (20.8%)
	Obsessive–compulsive disorder	4 (16.7%)
	Trauma-related disorder	4 (16.7%)
	Other disorder	6 (25.0%)

Note: * other was used where state or territory of residence *n* = 1, ** items were manually mapped against DSM-5 classifications and many participants reported multiple mental health diagnoses.

**Table 2:** A Summary of Themes.

Theme	Codes
3.2.1. The world is hard, and it’s getting harder	Complexity in friendship dynamics
	Pressure on young people: physically, socially and academically
	The world in crisis
	Lack of support and understanding from health professionals and caregivers
3.2.2. New media exacerbates old challenges	Access to self-harm communities has increased
	Awareness of and exposure to self-harm has increased
	Awareness of the world in crisis has increased
3.2.3. The online world brings unique challenges	Algorithm-driven social media platforms
	Exposure to self-harm narratives via streaming services
	The impact of the online world on mental health
	The online world can increase both risk and support

## Data Availability

The data presented in this study are available on request from the corresponding author due to ethical considerations.

## Supplementary Materials

The following supporting information can be downloaded at: https://www.mdpi.com/article/10.3390/ijerph22121871/s1. File S1: Interview Guide, File S2: COREQ (COnsolidated criteria for REporting Qualitative research) Checklist [64].
