# Supplementary material for: Perspectives from Young Australian Women with Lived Experience on Why Rates of Self-Harm Are Increasing: A Qualitative Study
Source: Int J Environ Res Public Health. 2025 Dec 16;22(12):1871. doi: 10.3390/ijerph22121871 (PMC12732825; doi:10.3390/ijerph22121871)
Supplement: Supplementary file 1 [file ijerph-22-01871-s001.zip › ijerph-3979543-supplementary File S1.pdf]

Supplementary S1 – Interview Guide

<u>Experience of Self-harm</u>

1. As far as you can remember, how old were you when you started thinking about self-harm?
2. How did you get the idea to self-harm initially?

   Prompts:

   - *School, social media, peers/friends/colleagues, mainstream media (e.g. TV, movies, books), family?*
3. Have you self-harmed on more than one occasion? If so, is there a time that you self-harmed which stands out to you?
   a. If yes, do you mind telling me about that experience?
   b. If no, okay, thinking about your experience of self-harm broadly then;

   Prompts (for both a and b):

   - *In the lead-up to this instance of self-harm, what was going on in your life?*
   - *What was going through your mind?*
   - *How were you feeling?*
   - *Where were you when you self-harmed?*
   - *What did self-harm look like? (what was the self-harm e.g. cutting)*
   - *How long had you been thinking about self-harming before you actually did self-harm?*
   - *Why did you pick that particular method?*
   - *What outcome were you hoping for at the time?*
     - o *Looking back, do you think there were other/different motivations you weren't aware of at the time?*
   - *How did you feel after self-harming? Was this how you expected to feel?*
4. [Skip if only one instance of self-harm]

   (If 'yes' to Q3.) Was this experience different to other experiences of self-harm? How so?

   (If 'no' to Q3) Does your experience of self-harm differ from one time to the next?

   Prompts:

   - *Are there similar triggers or situations that typically lead to you self-harming?*
   - *Did you use the same methods?*
   - *Is the time between thinking about self-harm and acting on it similar from one time to the next? (if 'yes' to Q3)*

- *Was your desired outcome the same in those instances to the one you just told me about?*
- *Did you feel differently or the same after those experiences in comparison to the one you just told me about?*

5. Have there been times when you thought about engaging in self-harm but didn't?

   If yes, what was different about those experiences to the experiences where you did self-harm?

   Prompts:

   - *Were the events leading up to those instances similar to the ones where you did self-harm?*
   - *Was there something in particular that stopped you? (e.g., thought, person, place)*

6. How long have you been engaging in self-harm? How has your experience of self-harm changed over time?

   Prompts:

   - *Has the frequency and severity changed?*
   - *Has your method of self-harm changed over time?*
   - *Has the reason/purpose for the self-harm changed over time?*
   - *Has the level of planning/impulsivity to self-harm changed over time?*
   - *If you tend to use self-harm as a way to cope/regulate emotions: Have you tried different coping strategies? What ways of coping other than self-harm have you found to be beneficial?*

<u>Help-seeking</u>

7. Is self-harm something you want/wanted to stop?

   Prompts:

   - *(If yes): why do you think you want/wanted to stop?*
   - *(if no): why do you think you don't/didn't want to stop?*

8. Did you ever tell anyone about your self-harm?

   a. If yes:

      - Who did you tell?
      - Why did you tell them?
      - How long after self-harming did you tell them?
      - How did they respond? Did their response affect your decision to tell others?

b. If no, is there a reason why you didn't tell anyone?

9. Have you sought help for self-harm?

   If yes, what was the nature of the help you sought? What has or has not worked for you? [*Prompts: help from family, friends, teachers, online, GP, mental health professionals? Other sources?*]

   If yes, was there anything that almost stopped you seeking help?

   If no, is there a reason why you haven't sought help? What would have made it easier for you to seek help?

10. What information or support would you have liked when you first started self-harming? How would you have liked to receive it?

    Prompts:

    - *Why do you feel that would be the best way to receive it?*
    - *When is the best way to receive it?*

11. Do you think there is anything that would have prevented you from starting to self-harm?

    Prompts:

    - *What information would you have liked to receive before you started self-harming?*
    - *How would you like to receive this information?*

12. Are there things that you think should be avoided when discussing self-harm, especially for young people? [*Prompts: language, type of information, ways of providing that information?*]

Closing out the interview

Introduction topic: We are coming to the end of the interview, I just have a few more questions.

- Research has shown that more young women are self-harming now than 10 years ago. Do you have any insights into why that would be?
- Do you have anything you would want to share with other young women who are engaging in self-harm or are thinking about it?
- Is there anything else you wanted to share about your experiences?
- Do you have any questions for me?
- **How do you feel about the interview experience?**
- **Would you like me to arrange for the clinical psychologist to call you in the next few days to check in and discuss referral options?**
- **Talking about self-harm can sometimes bring up difficult emotions. What is one thing that you can do for yourself after this interview as a form of self-care? (e.g. go for a walk, talk to a friend, listen to music, meditate etc.)**
